# Drug-Induced Epigenetic Changes Produce Drug Tolerance

**DOI:** 10.1371/journal.pbio.0050265

**Published:** 2007-10-16

**Authors:** Yan Wang, Harish R Krishnan, Alfredo Ghezzi, Jerry C. P Yin, Nigel S Atkinson

**Affiliations:** 1 Section of Neurobiology, The University of Texas at Austin, Austin, Texas, United States of America; 2 Institute for Neuroscience, The University of Texas at Austin, Austin, Texas, United States of America; 3 Department of Biology, Brandeis University, Waltham, Massachusetts, United States of America; 4 Department of Genetics, University of Wisconsin-Madison, Madison, Wisconsin, United States of America; 5 Department of Psychiatry, University of Wisconsin-Madison, Madison, Wisconsin, United States of America; 6 The Waggoner Center for Alcohol and Addiction Research, The University of Texas at Austin, Austin, Texas, United States of America; Salk Institute for Biological Studies, United States of America

## Abstract

Tolerance to drugs that affect neural activity is mediated, in part, by adaptive mechanisms that attempt to restore normal neural excitability. Changes in the expression of ion channel genes are thought to play an important role in these neural adaptations. The *slo* gene encodes the pore-forming subunit of BK-type Ca^2+^-activated K^+^ channels, which regulate many aspects of neural activity. Given that induction of *slo* gene expression plays an important role in the acquisition of tolerance to sedating drugs, we investigated the molecular mechanism of gene induction. Using chromatin immunoprecipitation followed by real-time PCR, we show that a single brief sedation with the anesthetic benzyl alcohol generates a spatiotemporal pattern of histone H4 acetylation across the *slo* promoter region. Inducing histone acetylation with a histone deacetylase inhibitor yields a similar pattern of changes in histone acetylation, up-regulates *slo* expression, and phenocopies tolerance in a *slo*-dependent manner. The cAMP response element binding protein (CREB) is an important transcription factor mediating experience-based neuroadaptations. The *slo* promoter region contains putative binding sites for the CREB transcription factor. Chromatin immunoprecipitation assays show that benzyl alcohol sedation enhances CREB binding within the *slo* promoter region. Furthermore, activation of a CREB dominant-negative transgene blocks benzyl alcohol–induced changes in histone acetylation within the *slo* promoter region, *slo* induction, and behavioral tolerance caused by benzyl alcohol sedation. These findings provide unique evidence that links molecular epigenetic histone modifications and transcriptional induction of an ion channel gene with a single behavioral event.

## Author Summary

A startlingly large number of adolescents abuse organic solvent inhalants, common components of glues, paints, and cleaning solutions. Our focus is on the molecular basis of tolerance—reduced response to a drug over time—which promotes increased drug consumption and accelerates the process of addiction. We use the fruit fly Drosophila melanogaster as a model system to determine how the nervous system becomes tolerant to the sedative effects of organic solvents. Sedating *Drosophila* with an organic solvent (benzyl alcohol) increases the expression of the *slo* K^+^ channel gene, which accelerates recovery from sedation. To elucidate the molecular mechanics underlying these phenomena, we documented dynamic changes in a chemical modification (called histone acetylation) that occurs within the *slo* regulatory region after sedation. These changes were mediated by a transcription factor and are linked to both *slo* induction and behavioral tolerance. Increased expression of *slo* channels is predicted to alter the signaling properties of neurons. This alteration, we propose, directly speeds the recovery from sedation.

## Abstract 3

Tolerance to solvents of abuse--at least in*Drosophila*--is due to epigenetic modification of the*slo* potassium channel gene.

## Introduction

Drug tolerance can be defined as reduced responsiveness to an effect of a drug caused by prior exposure to the drug [1]. With regard to recreational drugs, tolerance can cause an increase in self-administration and thereby speed the user down the path to addiction and/or to overdose. Most organic solvents are potent central nervous system depressants that produce sedation if inhaled or consumed in sufficient quantities. These properties have led to the use of such solvents both as anesthetics and as drugs of abuse. We have used the model system Drosophila melanogaster to study the neuronal basis of tolerance to organic solvent sedation.

When exposed to a small sedating dose of an organic solvent, such as benzyl alcohol (BA) or ethanol, *Drosophila* acquire rapid tolerance to subsequent solvent sedation. Rapid tolerance is defined as reduced drug responsiveness caused by a single prior exposure to the drug. In flies, this manifests itself as a reduction in the duration of sedation. Changes in the expression of the *slo* Ca^2+^-activated K^+^ channel gene have been linked to the production of rapid tolerance. It has been shown that sedation by a variety of methods induces *slo* gene expression in the nervous system, that *slo* mutations block the acquisition of behavioral tolerance, and finally, that transgenic induction of *slo* phenocopies the tolerant phenotype. Thus, the transcriptional regulation of the *slo* gene appears to be of general importance for the production of tolerance to sedation by various organic solvents [2–4]. We wish to understand how sedation with organic solvent anesthetics modulates the *slo* transcriptional control region. We continue to use BA as a model organic solvent anesthetic in this endeavor because it is well tolerated by flies and can be easily administered.

One of the first steps in transcriptional activation is commonly thought to be the alteration of chromatin structure. Specific amino acids in the N-termini of core histones can be modified by phosphorylation, acetylation, methylation, or ubiquitylation [5]. In particular, histone acetylation is believed to relax chromatin to make the DNA more accessible for recognition and binding by the transcriptional machinery [6]. Histone acetyltransferases (HATs) and histone deacetylases (HDACs) are enzymes that modulate histone acetylation states. Many transcription activators, such as cAMP-response element binding protein (CREB) binding protein (CBP)/p300, have HAT activity, and some transcription repressors, such as Sin3 and RPD3, have HDAC activity (for review, see [7]).

Recent studies indicate that histone acetylation contributes to the regulation of neural excitability and synaptic plasticity. It has been shown to have roles in learning and memory, in the production of circadian rhythms, and in the response to seizure, and has been identified as an important component underlying cocaine-induced neural plasticity [8–16]. Moreover, the administration of HDAC inhibitors has been shown to enhance long-term memory, arrest neurodegeneration, and alter cocaine responses [11,12,17,18].

Transcriptional regulation of the Drosophila slo channel gene is very complex. It has a 7-kb regulatory region that includes at least five transcriptional promoters that mediate developmental and tissue-specific gene expression. The two upstream promoters are neural specific. Neural expression is requisite for the acquisition of tolerance to both the anesthetic BA and to ethanol [2,4,19,20]. Within the *slo* transcriptional control region, there exist multiple putative CREB DNA binding sites. The history of CREB makes it an attractive candidate for the sedation-mediated activation of the *slo* gene and the production of tolerance.

CREB is a key transcription factor involved in regulating neural plasticity in response to various environmental stimuli [21]. Activated CREB binds to the *cis*-regulatory DNA element cAMP-response element (CRE) to induce target gene expression. In mammals, CREB has been implicated in the production of neuronal changes associated with drug tolerance and addiction. Exposure to addictive drugs, including opiates, cocaine, nicotine, and ethanol, induces CREB phosphorylation and CRE-mediated gene expression in the mouse nucleus accumbens (NAc), a major brain reward region [22–27]. The elevated CREB function appears to cause tolerance to and dependence on drugs of abuse. For instance, increased CREB function in the NAc reduces the sensitivity of mice to the rewarding effects of cocaine, whereas decreased CREB function induces cocaine sensitivity [28]. Although the CREB target genes that contribute to these specific effects are still not known, it has been shown that genes that encode some peptide neurotransmitters (NPY and CRF), neurotrophic factors (BDNF and IGF1), and even transcription factors (c-Fos) are downstream targets of CREB in specific brain regions [29–34]. However, these are thought to represent only a small subset of the genes regulated by CREB, and many CREB target genes have not been identified [30].

One way that CREB can stimulate transcription is by recruiting CBP, which is a HAT [35]. By inducing histone acetylation, CBP makes DNA more accessible to the transcriptional machinery [5]. It has been shown that the histone acetylation at c-Fos gene promoter, which is mediated by the recruitment of CBP, contributes to cocaine sensitization [36]. In *Drosophila*, the cAMP-PKA signaling pathway, a pathway activating CREB, has been implicated in the development of drug tolerance. *Drosophila* carrying a mutation in *amnesiac,* a gene encoding a neuropeptide that activates the cAMP pathway, show increased sensitivity to alcohol [37,38]. Conversely, fly mutants with increased PKA activity show decreased sensitivity to ethanol and cocaine [39].

Here, we show that a sedating dose of BA changes the chromatin acetylation state of the *slo* promoter region and that these changes are linked to increased *slo* expression, which produces behavioral tolerance to the sedative effect of BA. Furthermore, we show that the CREB transcription factor has an essential role in the production of acetylation changes, *slo* induction, and behavioral tolerance to organic solvent sedation.

## Results

### Benzyl Alcohol Sedation Dynamically Alters the Acetylation State of the *slo* Control Region

A strong link exists between gene induction and the acetylation of amino-terminal lysines in histone H4 in promoter regions [6,40,41]. We used the chromatin immunoprecipitation (ChromIP) assay to detect changes in histone H4 acetylation across the *slo* transcriptional control region, following BA sedation. The antibody used in ChromIP recognizes histone H4 acetylated at lysines K5, K8, K12, and K16 in *Drosophila* and mammals [10,42].

The amount of DNA associated with acetylated histone H4 was quantified by real-time PCR using primers specific for conserved portions of the *slo* transcriptional control region (4b, 6b, cre1, 55b, and cre2), the two neural promoters (C0 and C1), and the muscle promoter (C2) (Figure 1A). As a control, the histone acetylation levels at the promoter of the *Gpdh* gene were measured. Neither the *Gpdh* mRNA abundance nor the acetylation state of the *Gpdh* promoter was altered by BA sedation.

**Figure 1:**
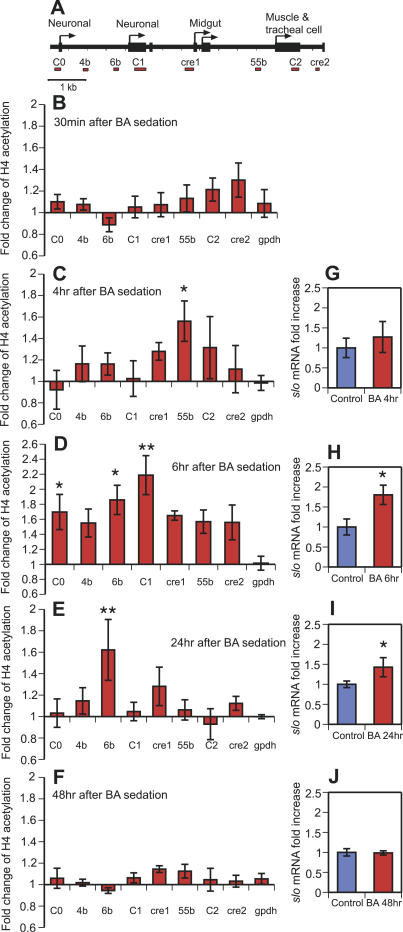
Benzyl Alcohol Sedation Causes Changes in the Acetylation Pattern of the *slo* Control Region (A) Map of the *slo* transcription control region. The *slo* gene has at least five tissue-specific promoters. The transcriptional start sites for these promoters are identified by the arrows. Closed boxes on the line represent exons. The row of boxes below the line represent evolutionarily conserved regions assayed in the ChromIP assay. (B–F) Acetylation state of histone H4 surveyed after BA sedation as determined by ChromIP and real-time PCR. Fold change is the ratio of acetylation levels obtained for BA sedated animals and mock-sedated age-matched controls. Bar graphs represent the mean ± SEM for three to six independent experiments. Statistical significance was determined by one-way ANOVA with Dunnett's comparison (*n* = 3–6, a single asterisk [*] indicates *p* < 0.05, and double asterisks [**] indicate *p* < 0.01 with respect to *Gpdh*). (B) There is no significant change in histone H4 acetylation 30 min after BA sedation. (C) Four hours after BA sedation, there is a significant increase in acetylation at conserved region 55b. (D) Six hours after BA sedation, hyperacetylation was observed over both neural promoters and 6b. (E) Twenty-four hours after BA sedation, the acetylation state of histone H4 has changed. Peak acetylation levels are centered at the conserved 6b region, and the level of acetylation of other regions has returned to control levels. (F) By 48 h, acetylation level across the *slo* promoter region has returned to baseline levels (no change relative to the mock-treated animals). (G–J) Relative *slo* mRNA abundance measured by real-time RT-PCR (using primers that recognize the transcripts produced from both neural promoters, C0 and C1) at 4, 6, 24, and 48 h, respectively, after BA sedation. Significance was determined using the Student *t*-test (*n* = 4; a single asterisk [*] indicates *p* < 0.05).

No significant change in histone acetylation was observed 30 min after BA sedation (Figure 1B). However, we observed a finely focused increase in histone H4 acetylation in the vicinity of 55b 4 h after BA sedation (Figure 1C). This acetylation change is likely to represent an early step in gene induction at which increased *slo* expression is not yet apparent (Figure 1G). The detection of increased message abundance may require time for the product to accumulate. Six hours after BA sedation, we observed a broad acetylation peak that included both neural promoter C0 and C1 (Figure 1D). This coincided with increased neural expression of *slo* (Figure 1H). By 24 h, *slo* expression appeared to be in decline (Figure 1I), and the enhanced histone H4 acetylation was again finely focused. However, this time, acetylation was centered over 6b, which is about 300 bp upstream of neural promoter C1 (Figure 1E). These data indicate that histone acetylation across the *slo* transcriptional control region is dynamically modulated after sedation with the anesthetic BA. By 48 h, the acetylation level and *slo* mRNA level have returned to the pretreatment level (Figure 1F and 1J).

### Increased Histone Acetylation Induces *slo* Gene Expression and Mimics Tolerance

To determine whether the observed changes in histone acetylation are related to *slo* induction and to the acquisition of BA sedation–induced BA tolerance, we used sodium butyrate to induce histone acetylation. Sodium butyrate is an extremely well-tolerated, nonspecific HDAC inhibitor that has been used to artificially induce histone acetylation both in mammals and insects [11,18,43–46]. Newly eclosed wild-type Canton S flies were split into two groups that differed only in the presence or absence of sodium butyrate in their food. Flies in the experimental group were fed food that contained 0.05 M sodium butyrate, and control group flies were fed food that lacked sodium butyrate. After 3 d, fly heads were collected, and ChromIP assays were performed to determine the acetylation state of histone H4. Overall histone H4 acetylation levels, expressed as the ratio of DNA associated with the acetylated chromatin to input DNA, were increased in the experimental group. As previously reported [18,42,43], sodium butyrate administration increased the global histone H4 acetylation level by approximately 30% (Figure 2A). The increase in acetylation is not uniform across the *slo* control region. Statistically significant increases in acetylation were observed only at region 6b (Figure 2B). Sodium butyrate, a nonspecific HDAC inhibitor, alters acetylation at the *slo* promoter region because it raises global histone acetylation levels. However, BA does not behave as a nonspecific enhancer of histone acetylation, because sedation with BA does not enhance global acetylation levels (Figure 2A).

**Figure 2:**
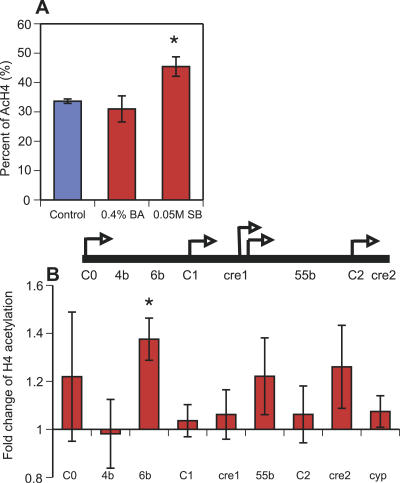
The HDAC Inhibitor Sodium Butyrate Enhances Histone Acetylation Levels (A) Sodium butyrate (SB) increases the global level of histone H4 acetylation. Control represents the global histone H4 acetylation level in flies fed normal food; 0.4% BA represents the level of histone H4 acetylation in flies sedated with 0.4% BA; and 0.05M SB represents the global histone H4 acetylation level in animals fed food supplemented with 0.05 M sodium butyrate (*n* = 4; a single asterisk [*] indicates *p* < 0.05, Student *t*-test). The H4 acetylation level is expressed as the percent ratio of the amount of DNA coimmunoprecipitated by anti-acetylated histone H4 antibody versus the total amount of input DNA used in the immunoprecipitation. (B) ChromIP assays indicate that the consumption of food containing 0.05 M sodium butyrate induced histone H4 acetylation specifically at the evolutionarily conserved 6b sequence of the *slo* transcriptional control region. Bar graphs represent the mean ± SEM for three to six independent experiments. Statistical significance was determined by one-way ANOVA with the Dunnett comparison (*n* = 3–6; a single asterisk [*] indicates *p* < 0.05, with respect to *cyclophilin 1*).

Our working hypothesis was that BA sedation increases histone acetylation, which in turn stimulates *slo* expression, and that this increase in *slo* expression leads to behavioral drug tolerance. In support of this hypothesis, we observed that sodium butyrate consumption both induced *slo* expression and phenocopied BA tolerance. As shown in Figure 3A, sedation with BA caused a 50% increase in neural *slo* mRNA abundance 6 h after sedation, whereas the consumption of sodium butyrate–laced food produced a 2-fold induction in neural *slo* messenger abundance (Figure 3A).

**Figure 3:**
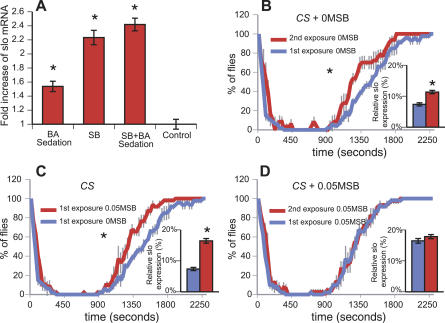
Sodium Butyrate Causes a Tolerant-Like Phenotype and Induces *slo* Levels (A) *slo* mRNA levels in Canton S (CS) heads were measured by real-time RT-PCR with C1 primers that only amplify neural *slo* transcripts. Six hours after BA sedation, *slo* mRNA increased approximately 50% compared with nonsedated flies. Sodium butyrate consumption induced *slo* expression about 2-fold. BA sedation of sodium butyrate–fed flies did not further increase *slo* mRNA abundance (*n* = 4; *p* < 0.05, Student *t*-test). (B) A tolerance assay showed that flies sedated with BA recover more rapidly after their second sedation than after their first sedation (24 h between sedations). Shown are recovery curves of a population of age-matched females after their first (blue) and their second (red) sedation. Plotted are the percentage of flies that have returned to wall climbing. Counts were made at 30-s intervals. The inset shows that the sedated population has higher neural expression of *slo* mRNA as measured by real-time PCR. (C) The curves show the recovery from BA sedation of flies that consumed food containing 0.05 M sodium butyrate (0.05MSB; red) and the recovery of flies that consumed the same food without sodium butyrate (0MSB;blue). The consumption of the HDAC inhibitor, sodium butyrate, mimics the tolerance phenotype shown in (B). The inset shows that neural expression of *slo* is increased by sodium butyrate consumption relative to the matched control. (D) Flies that had been fed sodium butyrate were sedated once (blue) or twice (red, 24 h between sedation) with BA. Both populations recovered from sedation at the same rate. The inset shows the mRNA expression level from *slo*. Notice that BA sedation plus sodium butyrate does not enhance *slo* expression more than sodium butyrate consumption alone. For all insets, *slo* mRNA abundance has been normalized to the *cyclophilin 1* internal control. For all plots, the significant differences between curves was determined by log-rank test (for [B], [C], and [D], *n* = 45; a single asterisk [*] indicates *p* < 0.05, and double asterisks [**] indicate *p* < 0.01).

A typical example of BA-induced behavioral tolerance is shown in Figure 3B. As previously described [2], flies recover more rapidly from their second sedation than from their first sedation. Figure 3C shows that the flies fed food laced with sodium butyrate exhibited a tolerance-like phenotype. Importantly, we observed that BA sedation of sodium butyrate–fed flies did not further enhance the expression level of *slo* (Figure 3A) or the relative degree of BA resistance (Figure 3D). This observation is consistent with the proposal that BA and sodium butyrate affect these characteristics through a common, saturable pathway.

### Sodium Butyrate–Induced Resistance Requires a Functional *slo* Gene

Sodium butyrate increases the level of acetylated histones globally [42,43]. Therefore, the tolerance-like phenotype caused by sodium butyrate consumption might be unrelated to the *slo* gene. To exclude this possibility, we determined whether *slo* mutations interfere with the capacity to acquire tolerance. The *slo* ^4^ allele is a loss-of-function *slo* mutation. It has been characterized as a null mutation genetically, molecularly, behaviorally, and electrophysiologically [47–49]. The *slo* ^4^ homozygous flies show subtle behavioral differences from wild-type flies, one of which is that they do not acquire tolerance following a single BA or ethanol sedation [2,3].

To test whether sodium butyrate–induced BA resistance was related to the *slo* gene, we asked whether the *slo* ^4^ mutation could block the capacity to acquire sodium butyrate–induced BA resistance. The consumption of sodium butyrate did not induce the tolerance-like phenotype in *slo* ^4^ homozygotes, but the wild-type Canton S flies, raised and tested under the same conditions, displayed the tolerance-like phenotype (Figure 4A and 4B). This indicates that the induction of BA resistance by sodium butyrate is dependent on a functional *slo* gene.

**Figure 4:**
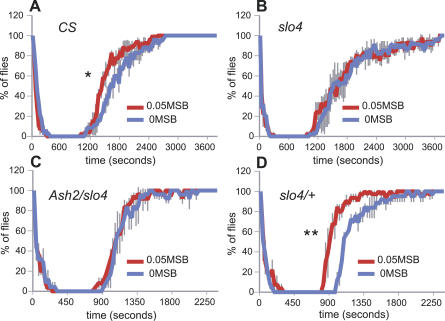
The Sodium Butyrate–Induced Benzyl Alcohol Resistance Phenotype Is Dependent upon a Functional *slo* Gene (A) The consumption of sodium butyrate causes wild-type Canton S (CS) flies to acquire BA resistance. The label 0MSB indicates flies fed normal food, and 0.05MSB represents flies fed food supplemented with 0.05 M sodium butyrate. (B) Sodium butyrate consumption does not cause *slo* ^4^ homozygotes to acquire BA resistance. (C) *ash2* ^18^/*slo* ^4^ transheterozygotes also did not show sodium butyrate–induced BA resistance. (D) However, *slo* ^4^/+ flies show a sodium butyrate–inducible resistance to BA sedation. The period of sedation in (A) and (B) versus (C) and (D) is somewhat different. This is likely the product of slight differences in the dose of the drug because the (A–B) pair and the (C–D) pair were performed on different days. Significance between recovery curves was determined by the log-rank test (*n* = 45; a single asterisk [*] indicates *p* < 0.05, and double asterisks [**] indicate *p* < 0.01).

Previous studies have indicated that only neural expression of *slo* is involved in the production of BA tolerance [2]. Therefore, we asked whether sodium butyrate–induced BA resistance could be blocked merely by eliminating neural expression of *slo*. The *ash2* ^18^ mutant chromosome carries a deletion that removes the two neural promoters of *slo,* but not the promoters responsible for expression in muscle, tracheal cell, or epithelia tissues. The deletion is a recessive lethal because it removes the neighboring gene [2,49,50]. Therefore, we used *ash2* ^18^/*slo* ^4^ transheterozygotes as a way to specifically eliminate *slo* expression in the nervous system. As shown in Figure 4C, the elimination of *slo* expression in the central nervous system prevents the induction of BA resistance by sodium butyrate. Figure 4D shows that sodium butyrate–induced BA resistance is a recessive phenotype of the *slo* ^4^ mutation.

### Benzyl Alcohol Sedation Induces dCREB2 Occupancy in the *slo* Transcriptional Control Region

In *Drosophila,* two CREB family genes have been identified, *dCREB-A* and *dCREB2* (also called *dCREB-B*). *dCREB-A* is weakly expressed in the adult brain [51] and has not yet been linked to behavioral phenotypes. The *dCREB2* gene is most similar to mammalian CREB and CREM genes and is considered to be the *Drosophila* homolog of these genes [52].

*dCREB2* is expressed in the adult brain, and has been implicated in the formation of learning, memory, and circadian behavior [52–54]. As with other transcription factors, CREB has functionally independent activation (KID) and DNA binding (bZIP) domains. In *Drosophila,* it has been shown that the KID domain of most dCREB2 proteins exists in a phosphorylated active state. As a result, in *Drosophila,* CREB activity has been postulated to be controlled at the level of DNA binding to its DNA elements [55]. Two putative CRE sites were identified close to 55b in the *slo* control region by sequence analysis. Furthermore, the highly conserved 55b element contains an AP-1 binding site motif. Both AP-1 and CREB belong to the b-ZIP family of transcription factors which share a similar DNA binding motif, the leucine zipper. The AP-1 response element (TGACTCA) is quite similar to the CREB response element, with only one base difference (CRE sequence: TGACGTCA). Both CREB and AP-1 can induce local histone acetylation by recruiting CBP HAT [35].

We performed the ChromIP assay using an anti-CREB antibody that recognizes the *Drosophila* dCREB2 DNA binding domain (Santa Cruz Biotechnology, http://www.scbt.com). We determined the relative occupancy of dCREB2 protein within the *slo* promoter region both before and after drug sedation. The data indicate a clear drug-induced increase in CREB binding at and around the 55b region (Figure 5).

**Figure 5:**
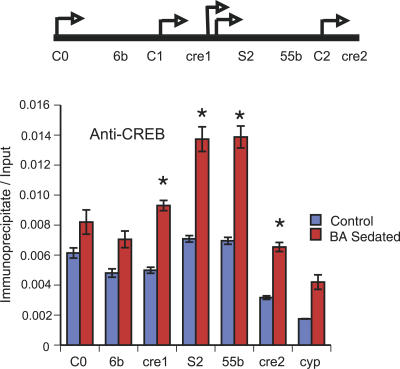
CREB Occupancy Is Enhanced by Sedation with Benzyl Alcohol Binding of CREB across the *slo* control region was measured 4 h after BA sedation with the ChromIP assay using an antibody against the CREB bZIP domain. Signals obtained from PCR amplification of immunoprecipitated chromatin were normalized to the signal from input chromatin. Increased CREB occupancy was observed at and between the two putative CRE sites called cre1 and cre2. Significance was determined by two-way ANOVA (*n* = 3; a single asterisk [*] indicates *p* < 0.05).

### Induction of a CREB Dominant-Negative Transgene Blocks Histone Acetylation within the *slo* Promoter Region

As previously described, BA sedation altered histone H4 acetylation across the *slo* promoter region. Shortly after sedation, acetylation was induced at 55b; a conserved site that is flanked by two CRE sites. It has been shown that binding of CREB at CRE sites can induce local histone acetylation by recruiting CBP and thereby activate gene expression [13,56]. To test whether the early acetylation peak over 55b within the *slo* control region is caused by the recruitment of CREB and CBP, we asked whether a dominant-negative CREB transcription factor could prevent the early histone acetylation peak at 55b. To do this, we transgenically expressed the repressor isoform of the dCREB2.

As in mammals, the dCREB2 transcript is alternatively spliced. The dCREB2a splice variant acts as a transcriptional activator, whereas the dCREB2b isoform has repressor activity [53]. A dominant-negative transgenic line that expresses dCREB2b under the control of a hsp70 heat-shock promoter (hs-dCREB2b) provided a way to inducibly inhibit CREB-mediated gene activation [54,57]. Previous studies have shown that a brief heat pulse (37 °C for 30 min) induces the transgene *(hs-dCREB2b)* and that elevated protein levels persist up to 24 h [54].

In this study, the *dCREB2b* transgene was induced, and histone acetylation levels were measured after BA sedation. These acetylation levels were compared to those from flies of the same genotype in which the transgene had not been activated. We observed that the induction of dominant-negative CREB by a brief heat pulse 1 h prior to BA sedation eliminated the histone acetylation peak around 55b (Figure 6). The same heat shock protocol has no effect on the histone acetylation changes at 55b in wild-type Canton S flies. This result suggested that the formation of an early acetylation peak requires the binding of CREB to CREs at *slo* transcriptional control region.

**Figure 6:**
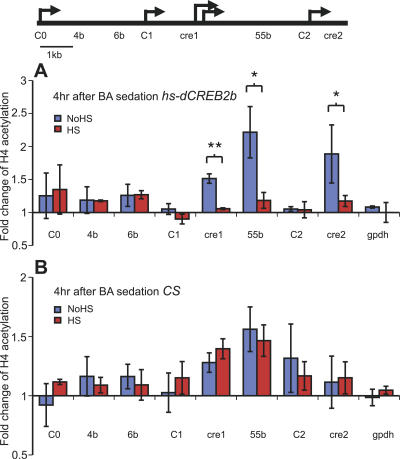
Induction of CREB Dominant-Negative Transgene Blocks Acetylation Changes Produced by Benzyl Alcohol Sedation The histone H4 acetylation profile across the *slo* control region was assayed by ChromIP and real-time PCR 4 h after BA sedation. Fold change is the ratio of H4 acetylation of sedated animals to mock-sedated control animals. (A) Without heat induction (NoHS; blue bars) of the dominant-negative CREB transgene *(hs-dCREB2b),* there are significant changes in H4 acetylation at cre1, 55b, and cre2. These changes in H4 acetylation were blocked by heat-pulse induction of *hs-dCREB2b* (HS; red bars) 1 h before BA sedation. (B) BA sedation of the Canton S (CS) stock (wild-type, nontransgenic) produces the same acetylation profile regardless of whether the stock had also been heat treated (HS; red bars) or not heat treated (NoHS; blue bars). The graph depicts data from four independent experiments (mean ± SEM). Statistical significance refers to the difference between the NoHS and HS treatments, and was determined by two-way ANOVA (*n* = 4; a single asterisk [*] indicates *p* < 0.05, and double asterisks [**] indicate *p* < 0.01).

### Activation of a Dominant-Negative CREB Transgene Reduces Benzyl Alcohol Sedation–Induced *slo* Expression

The previous data indicate that CREB activity is involved in the drug sedation–induced hyperacetylation within the *slo* promoter region. Acetylation changes have been linked to *slo* induction. Therefore, we asked whether dominant-negative CREB could interfere with the induction of *slo* after drug sedation. Both *hs-dCREB2b* transgenic females and wild-type CS females were subjected to 37 °C incubation for 30 min, 1 h before BA exposure, and the relative *slo* messenger levels were measured 6 h after drug exposure by real-time reverse transcriptase PCR (RT-PCR). We observed that induction of the dominant-negative CREB transgene 1 h prior to BA sedation suppressed *slo* induction (Figure 7). The same heat shock protocol did not suppress *slo* induction in wild-type CS flies following BA sedation. This result suggests that disruption of early histone acetylation pattern by inhibiting CREB activity blocks BA sedation–induced *slo* up-regulation.

**Figure 7:**
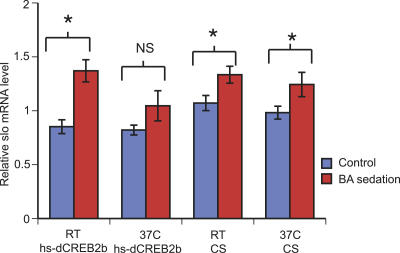
Activation of the Dominant-Negative CREB Transgene Blocks Benzyl Alcohol Sedation–Induced *slo* Expression Neuronal *slo* expression in *hs-dCREB2b* and Canton S (CS) wild-type fly heads were measured by real-time RT-PCR using C1 primers that are specific for neural *slo* transcripts. Blue bars represent the *slo* expression level in animals that were not sedated. Red bars show the level of *slo* mRNA expression 6 h after sedation. The *hs-dCREB2b* line shows robust *slo* induction after sedation if the transgene is not induced (RT *hs-dCREB2b*). Induction of the *hs-dCREB2b* transgene (37C hs-dCREB2b) reduces induction of *slo* by sedation. The transgene was induced with a 30 min/37 °C heat pulse 1 h before BA sedation. This treatment protocol does not affect the relative levels of *slo* expression in nontransgenic flies. Nontransgenic wild-type Canton S flies have similar levels of *slo* expression when they have been maintained at room temperature (RT CS) and when they have been subjected to the heat-induction protocol (37C CS). The relative *slo* mRNA levels were shown as ratio of messenger levels of *slo* to messenger levels of the *cyclophilin 1* gene, which functions as a internal control (*n* = 4; a single asterisk [*] indicates *p* < 0.05, Student *t*-test). NS, not significant.

### Induction of a Dominant-Negative CREB Transgene Prevents the Acquisition of Tolerance

The data above indicate that overexpression of dominant-negative dCREB2 can block drug-induced acetylation in the *slo* promoter region and the up-regulation of *slo* expression. Because *slo* induction appears causally linked to the production of tolerance in this behavioral paradigm, the activation of the *hs-dCREB2b* transgene should also block the acquisition of tolerance.

To test this idea, we examined induced and uninduced *hs-dCREB2b* transgenic flies for their capacity to acquire tolerance. Uninduced *hs-dCREB2b* flies acquired robust tolerance (Figure 8A). However, induction of *dCREB2b* transgene by heat shock 1 h before the first BA sedation completely blocked the acquisition of tolerance (Figure 8B). This occurred without a change in the initial sensitivity of flies to drug sedation (Figure 8C). The heat-activation protocol did not alter the relative magnitude of tolerance acquired by a wild-type Canton S stock (Figure 8D and 8E). These data indicate that CREB transcription factor activity is important for the acquisition of rapid tolerance in flies.

**Figure 8:**
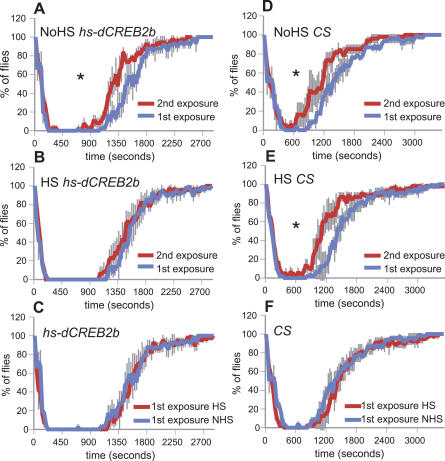
Induction of the Dominant-Negative CREB Transgene Prevents the Acquisition of Tolerance Shown are recovery curves of a populations of age-matched females after their first (blue) and their second (red) BA sedation. Plotted are the percentage of flies that have returned to wall climbing. Counts were made at 30-s intervals. (A) *hs-dCREB2b* transgenic flies developed rapid tolerance if the transgene is not induced (no heat shock [noHS]). (B) Induction of the *dCREB2b* transgene by a brief heat shock (37 °C, 30 min; HS) 1 h before the first sedation blocked the acquisition of rapid tolerance, which is measured 24 h after initial drug sedation. (C) Heat shock had no effect on the wake-up time of *hs-dCREB2b* transgenic flies from their first sedation. (D and E) Wild-type Canton S control group developed rapid tolerance after first sedation regardless of whether they were subjected to the heat protocol. (F) Heat shock by itself had no effect on the wake-up time of CS flies from their first sedation. Significance between recovery curves was determined by log-rank test (*n* = 45; a single asterisk [*] indicates *p* < 0.05).

## Discussion

It has become clear that drug-induced changes in gene expression play an important role in the pharmacodynamic response to drugs of abuse. Epigenetic modifications to promoter regions are rapidly emerging as an important mechanism for producing these changes [11,58]. Epigenetics refers to DNA and chromatin modifications that influence chromatin structure and change the state of gene expression without altering the nucleotide sequence [59]. Hyperacetylation of histone H4 has been linked to neural gene activation in activity-dependent signaling pathways and in synaptic plasticity [10,13]. In *Drosophila,* sedation with BA induces neural *slo* expression in a dose-dependent manner, and increased *slo* gene expression has been implicated in the production of rapid tolerance to both ethanol and BA [2–4].

An increase in histone acetylation is generally accepted to make DNA sequences more available to transcription factors. Hyperacetylation at one site might beget subsequent hyperacetylation at a second site. That is, the activity of one transcription factor could modify an area to facilitate the binding of additional transcription factors. The action of these factors may continue to further modify the region. We show that sedation with the anesthetic BA produces a specific spatiotemporal pattern of histone H4 acetylation across the *slo* promoter region. These epigenetic changes are correlated with changes in *slo* transcription and with the development of drug tolerance, and are thought to be the molecular footprints of a regulatory cascade that is initiated by BA sedation.

The acetylation spike at 55b occurs immediately prior to the induction of *slo* expression and may make available specific sequences that are necessary to activate the upstream neural promoters. Two hours later, we recorded the greatest increase in *slo* expression and an increase in acetylation that involves most of the *slo* neural promoter region. The process of transcription itself can enhance acetylation, and therefore, this broad boost in acetylation may be a direct by-product of transcription [60]. Finally, at 24 h post BA sedation, there is an isolated acetylation spike at 6b, and *slo* expression remains elevated (albeit slightly less elevated than at 6 h post sedation). This may mean that increased accessibility of 6b is required to maintain *slo* induction. We propose that this dynamic pattern of hyperacetylation represents an unfolding regulatory program that leads to a transient and self-limiting boost in *slo* channel expression.

We used the HDAC inhibitor sodium butyrate to artificially induce histone acetylation in the genome. We asked whether a simple change in acetylation would induce *slo* gene expression and whether it could also produce a tolerance-like phenotype. We observed that sodium butyrate consumption induced *slo* expression and phenocopied the BA sedation–induced tolerance phenotype. In various preparations, sodium butyrate has been shown to enhance histone acetylation and to induce expression of a small fraction of the genes in the eukaryotic genome (estimates range from 1% to 7%) [45,61,62]. Therefore, our result would be irrelevant if we could not directly link sodium butyrate–induced BA resistance to *slo,* and demonstrate other similarities to the effects of BA sedation. Using *slo* null mutations, we were able to show that the capacity of sodium butyrate to phenocopy tolerance is dependent on *slo* expression in the nervous system, as is rapid tolerance induced by BA or ethanol sedation [2,3]. In addition, BA sedation of sodium butyrate–fed flies did not further enhance resistance, suggesting that both act through a common, saturable pathway. Finally, we were surprised to find some similarity between sodium butyrate and BA sedation upon the pattern of acetylation on the *slo* transcription control region. Sodium butyrate also caused hyperacetylation at the conserved 6b sequence, but did not detectably change acetylation at C1, cre1, or 55b sites. This mimics the acetylation pattern observed 24 h after BA sedation. Since sodium butyrate inhibits HDAC activity, the enhancement of 6b acetylation by sodium butyrate suggests that it has inhibited an HDAC that is chronically positioned near or at the 6b site. The simplest interpretation is that enhanced availability of 6b permits *slo* induction.

Both 55b and 6b are non–promoter-containing DNA sequences that were originally identified because of their conservation between two *Drosophila* species [19,63]. Conservation alone is a strong indicator that these sequences have important roles in the regulation of the *slo* gene. Figure 9 shows that they are highly conserved across at least eight *Drosophila* species.

**Figure 9:**
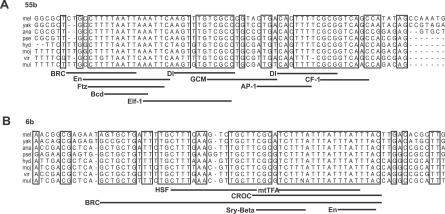
Genomic Sequence Conservation of 55b and 6b across Multiple Species of *Drosophila* (A) Alignment of the 55b box [63] and (B) the 6b box [19] across eight *Drosophila* species. Areas of highest identity (80%) are boxed, and black bars below the line denote transcription factor binding motifs that are conserved in these species. *Drosophila* species: ana, *D. ananassae;* hyd, *D. hydei;* mel, *D. melanogaster;* moj, *D. mojavensis;* mul, *D. mulleri;* pse, *D. pseudoobscura;* vir, *D. virilis;* and yak, D. yakuba. Transcription factor binding sites: AP-1, Activator Protein-1; Bcd, Bicoid; BRC, Broad Complex; CF-1, Chorion Factor 1; CROC, Crocodile; DL, Dorsal; Elf-1, Element I-binding activity; En, Engrailed; Ftz, Fushi Tarazu; GCM, Glial Cells Missing; HSF, Heat Shock Motif; mtTFA, Mitochondrial Factor A; and Sry-Beta, Serendipity Beta.

Two putative CREB binding sites are located close to 55b. The cre1 site is TGACGAA and cre2 is TGACGTAA. The cre1 site matches the canonical CRE motif, whereas cre2 differs in a single nucleotide. Both include the first five bases of the consensus CRE sites (TGACG), which are sufficient for CREB-mediated transcription [64]. Furthermore, in 55b, there is a putative AP-1 site (TGATTCA) which differs in two nucleotides from the canonical CRE site. AP-1 and CREB carry similar dimerization and DNA binding b-ZIP domains. Studies suggest AP-1 and CREB can form cross-family heterodimers and share same consensus DNA binding elements [65].

The AP-1 and CREB transcription factors have been implicated in the neural response to abused drugs. AP-1 transcription factor complexes are dimers formed from the Jun and Fos family of transcription factors. Acute administration of certain abused drugs causes complex changes in the pattern of the Fos family members [27,66–68]. These changes affect drug responsiveness and are thought to underlie long-lasting sensitization to cocaine in mammals [67]. The modulation of CREB activity has been linked to drug tolerance and dependence and many other neural responses that can all be considered to be forms of neural plasticity [67,69–71].

Both AP-1 and CREB can stimulate gene expression by recruiting cofactor CBP, which helps to position the basal transcriptional machinery [35,72]. CBP is a HAT that through histone acetylation causes local decondensation of chromatin [35]. We postulated that AP-1 or CREB might be involved in acetylation at 55b. ChromIP experiments indicated that AP-1 did not bind the *slo* promoter region (unpublished data). However, ChromIP did show that CREB bound in this region and that drug sedation enhanced CREB occupancy. We further implicated CREB, in drug-mediated *slo* expression and behavioral tolerance, by showing that a dominant-negative CREB transcription factor could block hyperacetylation surrounding 55b, the induction of *slo,* and behavioral tolerance caused by BA sedation.

These data suggest a chain of events for the regulation of *slo* in response to BA sedation. That is; drug sedation activates the CREB signaling pathway. CREB binds the CRE sites flanking 55b and perhaps within 55b itself, and then recruits the CBP HAT to acetylate histones in the neighborhood of 55b. This makes sequences at 55b available for binding by factors that lead to further modifications and result in the inhibition of the HDAC positioned at 6b. Inhibition of this HDAC augments 6b acetylation, making it available for binding by a factor (perhaps HSF, Figure 9) that directly stimulates expression from the two neighboring neural promoters. Blockage of any of these events would interfere with the drug-induced *slo* expression and animals' ability to develop tolerance. Although other more complex models are possible, this simple model will eventually be testable as new tools become available, and is useful for organizing ideas about how *slo* senses and responds to drug sedation.

We have previously postulated that the induction of *slo* is a homeostatic response that acts to reverse decreased neural excitability associated with sedation by the drug BA. Anesthesia induces *slo* gene expression, and by itself, the induction of this channel gene phenocopies tolerance [2]. Our data are consistent with the idea that increased BK channel expression reduced the duration of sedation caused by BA and produced a tolerance-like phenotype. This is an unusual role to postulate for a K^+^ channel. Certainly, in some preparations, increased BK channel activity reduces neural excitability [73–75]. However, in other preparations, BK channel activity has been positively correlated with neural excitability [76–80]. It has been proposed that an increase in BK channel activity limits the instantaneous response of the cell, but augments the capacity for repetitive neural activity by reducing the neural refractory period [76,77]. The refractory period is the time that must elapse before the neuron can fire again.

Neural pharmacodynamic tolerance to any drug is likely to involve many components [81–84]; however, the *slo* gene is uniquely positioned to be a homeostatic regulator of neural excitability. The encoded channel has the highest conductance of any neural ion channel, thus small changes in its density can have a large influence on membrane excitability. In conclusion, we propose that in flies, BA sedation causes CREB-mediated epigenetic changes in the *slo* control region that result in an increase in *slo* expression, which significantly enhances the excitability of the nervous system to help produce the tolerance phenotype.

## Materials and Methods

### Fly stocks.

*Drosophila* stocks were Canton S (wild type), *slo* ^4^, *ash2* ^18^/*slo* ^4^, and the *hs-dCREB2b.* All fly stocks were raised on standard cornmeal agar medium (12/12-h light/dark cycle). Newly eclosed flies collected over a 1- to 2-d interval were studied 4–5 d after eclosion. The *hs-dCREB2b* transgene expresses a *dCREB2b* cDNA from a hsp70 promoter [54].

### Benzyl alcohol exposure and sodium butyrate consumption.

Age-matched female Canton S flies were divided into six groups of 15. Three groups were sedated with 0.4% BA, and three were mock sedated as previously described [2]. Sodium butyrate (0.05 M, 99% purity; Fisher Scientific, https://www.fishersci.com) in food was fed to 1- to 2-d-old flies for 3 d. Controls were fed unadulterated food.

### Tolerance assay.

Tolerance was measured as described [2]. In the first exposure, three experimental groups were exposed to 0.4% benzyl alcohol until sedation, and three control groups were mock sedated in parallel. Twenty-four hours later, all groups were sedated with BA, then transferred to an anesthetic-free environment, and recovered flies were counted every 30 s. Flies were scored as recovered when they resumed climbing. The log-rank test was used to determine significance between curves [85]. However, error bars represent the standard error of the mean (SEM) for each point.

### Chromatin immunoprecipitation assay.

About 1,500 wild-type flies were either BA sedated or mock sedated for 5 min and then were allowed to recover in a BA-free environment [2]. Thirty minutes, and 4, 6, 24, and 48 h after sedation, flies were collected, frozen in liquid nitrogen, and vortex decapitated, and then heads were collected by sieving. Heads were cross-linked with 2% formaldehyde for 5 min, and chromatin was solubilized and sonicated on ice 6 × 30 s, followed by 1 min cooling on ice to produce fragments of approximately 600 bp with a sonic Dismembrator 250 (Fisher Scientific) as described by Orlando et al. [86]. Sheared soluble chromatin was stored at −80 °C.

The ChromIP assay was performed as described (ChIP kit # 17–295; Upstate Biotechnology, http://www.upstate.com) with minor modifications. One milliliter of soluble chromatin (1 mg/ml) was adjusted to RIPA buffer and then precleared with 50-μl salmon sperm DNA/protein A agarose slurry for 1 h at 4 °C to reduce nonspecific binding. Ten percent of the preimmunoprecipitation lysate (100 μl) was held back and used to determine the input of DNA. The input-level control was processed with the eluted immunoprecipitations (IPs), beginning with the cross-linking reversal step. The polyclonal antibody (catalog # 06–866; Upstate Biotechnology) against acetylated H4 at K5, K8, K12, and K16, and anti-CREB antibody (sc-186; Santa Cruz Biotechnology) were used at 1:200 dilution. Five microliters of antibody were added to each sample in 1-ml RIPA buffer and incubated overnight at 4 °C with gentle mixing. Immunocomplexes were recovered by adding 80 μl of the salmon sperm DNA/protein A agarose beads, incubating for 3 h at 4 °C with rotation. The beads were sequentially washed three times in RIPA (140 mM NaCl, 1 mM EDTA, 10 mM Tris-HCl [pH 8], 1% Triton X-100, 0.1% SDS, 0.1% sodium deoxycolate), twice in high-salt buffer, once in LiCl buffer, and twice in TE buffer, 10 min each. The cross-linking between histones and DNA was reversed by incubating at 65 °C overnight, and DNA fragments were purified with phenol-chloroform extraction followed by acid ethanol precipitation. ChromIP assays were performed more than three times with independent tissue samples.

Real-time PCR was performed using the ABI SYBR Green PCR protocol (Applied Biosystems, http://www.appliedbiosystems.com). Within the *slo* transcriptional control region (Figure 1A), primers were designed to amplify approximately 200-bp fragments at the two neural promoters (C0 and C1), at one muscle promoter (C2), and at six evolutionarily conserved areas (4b, 6b, cre1, 55b, S2, and cre2). As internal controls, we used Gpdh (Glycerol-3-phosphate dehydrogenase) and Cyp1 (cyclophilin 1). Primers sets are: C0 (5′-ATCGAACGAAGCGTCCAG-3′, 5′-CGACGCGCTCAAACG-3′), 4b (5′-GACCCGATGATAAAGTCGATGT-3′, 5′-GCCAGTGACTGACTGACACACA-3′), 6b (5′-CCAGCAGCAATTGTGAGAAA-3′, 5′-CGAAGCAGACTTGAAAGCAA-3′), C1 (5′-ACAAACCAAAACGCACAATG-3′, 5′-AATGGATGAAGGACTGGGAGT-3′), cre1 (5′-GATGGGAAAGCGAAAAGACAT-3′, 5′-CATGTCCGTCAAAGCGAAAC-3′), 55b (5′-TACCCAATTGAATTCGCCTTGTCTT-3′, 5′-CCCACTCTCCGGCCATCTCT-3′), S2 (5′-CATTGCTATCCCTTCCCATC-3′, 5′-ATGCAATGAAGCGAAGAACC-3′), C2 (5′-GCACTCGACTGCACTTGAAC-3′, 5′-AATGAAAAAGTTCTCTCTGTGCAT-3′), cre2 (5′-TGGATTGCGACCGAGTGTCT-3′, 5′-ATCAATACGATAACTGGCGGAAACA-3′), Gpdh (5′-GCATACCTTGATCTTGGCCGT-3′, 5′-GCCCTGAAAAGTGCAAGAAG-3′), and Cyp1 (5′-TCTGCGTATGTGTGGCTCAT-3′, 5′-TACAGAACTCGCGCATTCAC-3′).

All amplicons have differences in standard-curve amplification slopes of less than 0.1. Melting curves were used to detect nonspecific amplification. Amplifications were run in triplicate, and the changes on histone H4 acetylation were calculated by the ΔΔCT method. Fold enrichment over control equals 2 ^ (CT^Input^ – CT^IP^)_experiment_/2 ^ (CT^input^ – CT^IP^)_control_. The entire protocol was repeated in triplicate a minimum of three times, and the mean and SEM were calculated. Significance was determined by one-way ANOVA.

For ChromIP to measure CREB binding, all real-time PCR measurement values were normalized to input DNA in both the BA-sedated and mock-sedated control. The amount of DNA recovered in the IP was expressed as the ratio of input DNA with the equation: IP/input = 2 ^ (Ct^input^ − Ct^IP^). The entire protocol has been repeated in triplicate, and the mean and SEM calculated. Significance was determined with the two-way ANOVA.

### Global acetylation assay.

Chromatin from cross-linked fly heads was sonicated and immunoprecipitated with anti-H4 antibody as described above. DNA coimmunoprecipitated with acetylated histone H4 and input DNA were purified by reverse cross-linking followed by phenol-chloroform extraction. DNA was then quantified in a NanoDrop spectophotometer (NanoDrop Technologies, http://www.nanodrop.com). Global histone H4 acetylation levels were expressed as the ratio of the amount of DNA associated with the acetylated chromatin to input DNA. The entire protocol has been repeated four times, and the mean and SEM calculated. Significance was determined with the Student *t*-test.

### Quantitative RT-PCR analysis.

RNA was isolated from heads using a single-step RNA isolation protocol as described previously [2] and quantified (NanoDrop Technologies). Reverse transcription and real-time RT-PCR were performed in triplicate with *slo* exon C1-specific primers, which only amplify transcripts from the neural promoters, and cyclophilin 1 primers as described [4]. Fold change was calculated using the standard-curve method (Applied Biosystems manual). Significance was calculated using the Student *t*-test.

Heat-induction protocol for the HS-dCREB2b transgene dCREB repressor (dCREB2b) transgene was induced at 37 °C for 30 min in a humidified incubator as described [54]. Immediately after heat shock, flies were sedated with CO_2_ to reduce the net effect of heat on the neural activity. Half an hour later, flies were stabilized and ready for tolerance assay. Mock induction was performed with Canton S flies (wild type).

## Supporting Information

### Accession Numbers

The National Center for Biotechnology Information (NCBI; http://www.ncbi.nlm.nih.gov) accession numbers for genes mentioned in this paper are (32595), *dCREB-A* (39682), *dCREB2* (32817), *Gpdh* (33824), and *slo* (42940).

## Abbreviations

BA: benzyl alcohol
ChromIP: chromatin immunoprecipitation
CRE: cAMP response element
CREB: cAMP response element binding protein
CBP: cAMP-response element binding protein (CREB) binding protein
*Cyp,*: cyclophilin 1
*Gpdh,*: glycerol-3-phosphate dehydrogenase
HAT: histone acetyltransferase
HDAC: histone deacetylase
RT-PCR: reverse transcriptase PCR
SEM: standard error of the mean
